# Long-Term Treatment with Bulevirtide in Patients with Chronic Hepatitis D and Advanced Chronic Liver Disease

**DOI:** 10.1155/2024/2364031

**Published:** 2024-07-23

**Authors:** Ayaz Sapuk, Leonie Steinhoff, Kristin Hünninghaus, Katharina Willuweit, Jassin Rashidi Alavijeh, Benedikt Hild, Lucia Asar, Hartmut H. Schmidt, Christoph Schramm

**Affiliations:** ^1^Department of Gastroenterology, Hepatology and Transplantational Medicine, University Hospital Essen, and Faculty of Medicine, University of Duisburg-Essen, Essen, Germany; ^2^Institute for Virology, University Hospital Essen, and Faculty of Medicine, University of Duisburg-Essen, Essen, Germany

## Abstract

Bulevirtide (BLV) is approved for the treatment of chronic hepatitis D (CHD). Because only limited long-term experience has been reported, we aimed to evaluate the efficacy and safety of BLV treatment in patients with advanced chronic liver disease (ACLD). We performed a retrospective analysis of patients with CHD who received BLV 2 mg/day for >12 months at a tertiary center. Virological response (VR) was defined as a reduction in hepatitis delta virus-ribonucleic acid (HDV-RNA) ≥2 log_10_ from baseline or HDV-RNA negativity and biochemical response (BR) as gender-specific normalization of transaminases. We identified 14 patients (9 men, 5 women; median age of 48 years; interquartile range (IQR) of 37–55), of whom 12 (86%) had suggested or assumed ACLD according to Baveno VI criteria. The median duration of BLV treatment was 26 months (IQR 17–27). During treatment, the mean HDV-RNA level decreased from log_10_ 5.58 IU/ml to levels between log_10_ 2.19 IU/ml and log_10_ 3.19 IU/ml. HDV-RNA negativity was achieved in up to 63% after 24 months. VR and BR were 86% and 43% after 12 months, 90% and 60% after 18 months, 75% and 75% after 24 months, and 100% and 50% after 30 months, respectively. Two nonpersisting viral breakthroughs were observed after 24 months of treatment. The Child Pugh score and model of end-stage liver disease (MELD) scores remained stable or improved in 12 patients (86%). Only one patient developed hepatic decompensation after 24 months of treatment with ascites requiring large-volume paracentesis which was not associated with viral breakthrough, portal vein thrombosis, or hepatocellular carcinoma. Treatment with BLV beyond one year is effective and safe for patients with CHD and ACLD. Liver function remained stable or improved during treatment in the vast majority of patients, and only one case of hepatic decompensation occurred during a median follow-up of 26 months.

## 1. Introduction

An estimated 12 million people are seropositive for hepatitis delta virus (HDV) worldwide, accounting for a global prevalence of 0.16% [1]. Among hepatitis B surface antigen (HBsAg)-positive people, the anti-HDV prevalence ranges between 3.0% in Europe and 6.0% in Africa, with an overall estimate of 4.5% [1]. In Germany, an incidence of 0.07 per 100,000 in the general population was reported. Around one third of patients with newly diagnosed HDV are not of German descent but originate mostly from Eastern Europe [2]. People from HDV-endemic areas, people who inject drugs, hemodialysis recipients, men who have sex with men, commercial sex workers, and individuals infected with hepatitis C virus or human immunodeficiency virus carry an increased risk of infection with HDV [1]. Despite its relatively low prevalence in HBsAg-positive patients, chronic hepatitis D (CHD) accounts for 18% of cases with cirrhosis and 20% of cases with hepatocellular carcinoma (HCC) emphasizing the aggressive course of the disease [1]. Patients with CHD tend to have more severe liver disease, more rapid progression to cirrhosis, and an increased risk for hepatic decompensation and death compared to patients with hepatitis B virus (HBV) monoinfection [3]. The term compensated advanced chronic liver disease (cACLD) was recently introduced by the Baveno VI consensus report, taking into account the difficulties of differentiating between severe fibrosis and cirrhosis by means of clinical evaluation or non-invasive testing [4].

HDV is a small, defective ribonucleic acid (RNA)-virus which can propagate only with coexisting HBV since it depends on HBsAg for its assembly [3]. HBsAg interacts with the sodium taurocholate cotransporting polypeptide (NTCP), a sodium-dependent uptake transporter localized on the basolateral membrane of hepatocytes responsible for uptake of bile acids, which facilitates the entry of HDV into hepatocytes [5]. Treatment options for CHD are limited and pegylated interferon alfa (PEG-IFN*α*) used to be the only treatment option, other agents such as nucleos(t)ide-analogue (NA) are ineffective despite its efficacy in chronic hepatitis B [6–8].

Bulevirtide (BLV), a large lipopeptide comprising 47 amino acids, is a first-in-class inhibitor of NTCP [5, 9]. It mimics a region of the pre-S1 HBsAg and inhibits viral entry by inhibitory competition [9]. It is fully approved for the treatment of CHD in HDV-RNA positive adult patients with compensated liver disease at a daily dose of 2 mg by the European Medicines Agency (EMA) after conditional approval in July 2020 based on the results from clinical trials [10–13]. According to the summary of product characteristics, treatment should be continued as long as it is associated with a clinical benefit as the optimal treatment duration is unknown [14]. Discontinuation should be considered in case of sustained HBsAg seroconversion or loss of virological and/or biochemical responses [14]. Randomized controlled trials investigating BLV reported promising results regarding its efficacy and safety, however, for limited treatment durations of 24 or 48 weeks [10–12]. Data on long-term (>48 weeks) administration of BLV are sparse.

The objective of this study was to evaluate the treatment course of adult patients with advanced chronic liver disease (ACLD) who received treatment with BLV for at least one year in a real-world setting.

## 2. Materials and Methods

### 2.1. Patients

We conducted a retrospective analysis of all patients with CHD who received BLV treatment at a dose of 2 mg per day according to the current label for >12 months at a tertiary academic center in Germany. Exclusion criteria were concomitant chronic liver disease of other etiologies, treatment with BLV within clinical studies or early access programs, and active hepatocellular carcinoma. Baseline was defined as the initiation of BLV treatment. During treatment, patients were scheduled for outpatient visits every 3 months.

### 2.2. Definitions

In accordance with clinical trials in HDV, the following definitions were applied [11]: virological response (VR) as reduction in HDV-RNA ≥2 log_10_ from baseline or HDV-RNA undetectability; biochemical response (BR) as normalization of transaminases, corresponding to alanine aminotransferase (ALT) <35 U/l in female and <50 U/l in male; combined response (CR) as the presence of VR and BR; partial response (PR) as reduction in HDV-RNA of ≥1 log_10_ and <2 log_10_ from baseline; non-response (NR) as reduction in HDV-RNA of <1 log_10_ from baseline; and virological breakthrough (VB) as increase in HDV-RNA of >1 log_10_ above nadir. Compensated advanced chronic liver disease was diagnosed via measurement of liver stiffness >15 kPa, or the presence of gastroesophageal varices in upper gastrointestinal endoscopy or severe fibrosis or cirrhosis on liver histology, according to the Baveno VI consensus report [3]. A liver stiffness 10–15 kPa was suggestive of cACLD [4]. Platelet count <150.000/*µ*l was defined as thrombocytopenia.

### 2.3. Scores and Techniques

Child Pugh score, model of end-stage liver disease (MELD) score, aspartate aminotransferase (AST)-to-platelet-ratio-index (APRI), fibrosis (FIB)4-score, Delta-4 fibrosis score (D4FS), and baseline-event anticipation (BEA) score were calculated as previously published [15–17]. Liver stiffness was measured by transient elastography (FibroScan®) according to current recommendations [15]. HDV-RNA levels were quantified with the RoboGene HDV-RNA quantification kit 2.0 (Roboscreen GmbH, Leipzig, Germany) using Rotor-Gene Q (Qiagen GmbH, Hilden, Germany). The linear range for measurement of HDV-RNA is 150 IU/ml to 15,000,000 IU/ml with a lower limit of detection (LLD) of 60 IU/ml and a lower limit of quantification (LLQ) of 150 IU/ml.

### 2.4. Statistics

Statistical analysis was performed using Statistical Package for the Social Sciences statistics version 28 (IBM, Chicago, USA) and MS Excel (Microsoft, Richmond, USA). Categorical variables were analyzed as absolute numbers and their relative frequencies and continuous variables as mean and standard deviation (SD) or median and interquartile range (IQR). Statistical significance was set at *P* < 0.05.

In accordance with German law, approval by a local ethics committee was not required (paragraph 15, sentence 1, North Rhine Medical Association's professional code of conduct from 14 November 1998 as amended on 19 November 2011), neither was a written informed consent obtained from the participants because of the strict retrospective design of our study (paragraph 6, sentence 1, Health Data Protection Act of North Rhine-Westphalia). Informed consent with patients was obtained prior to treatment initiation and BLV was prescribed according to the summary of product characteristics [14].

## 3. Results

### 3.1. Baseline Characteristics of Study Cohort

We identified 14 patients (9 men and 5 women) with a median age of 48 years (IQR 37–55) according to the inclusion and exclusion criteria above. Compensated ACLD at baseline was assumed in ten (71%) patients and was suggested in 2 (14%) patients, including 2 (14%) patients with a history of hepatic decompensation. The mean liver stiffness at baseline was 17.1 kPa (SD 7.4). One patient (7%) had FIB-4 <1.45, 3 (21%) patients had FIB-4 of 1.45–3.25, of whom one had a liver stiffness of 22.7 kPa, and 10 (71%) patients had FIB-4 >3.25. One patient (8%) had a D4FS >7.8. Splenomegaly and thrombocytopenia were present in 11 (79%) and 12 (86%) patients, respectively; 5 (36%) patients had esophageal varices, and 1 (7%) patient had diuretic-controlled ascites. All patients received concomitant NA therapy, and 6 (43%) patients had prior IFN therapy (median time of treatment 12 months and median time between IFN and BLV treatment 98.5 months). At baseline, the mean HDV-RNA was log_10_ 5.58 IU/ml (SD 1.08), and 11 (79%) patients had HBV-DNA below the LLQ with detectable HBV-DNA levels ranging from 12 to 20 IU/ml. The baseline characteristics of the patients are displayed in Table 1.

### 3.2. Response to Bulevirtid Treatment

**Figure 1:**
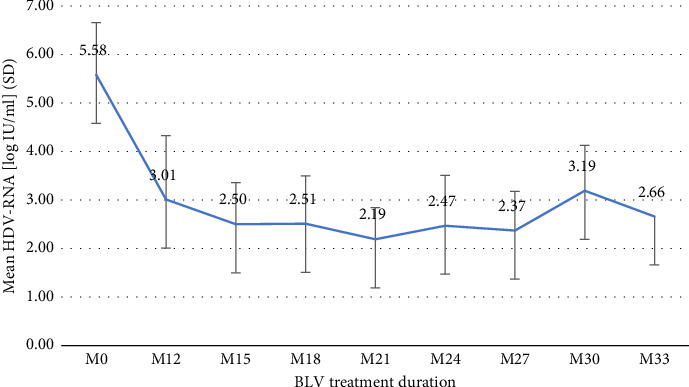
Virological response. SD: standard deviation; M: treatment months; BLV: bulevirtide.

**Figure 2:**
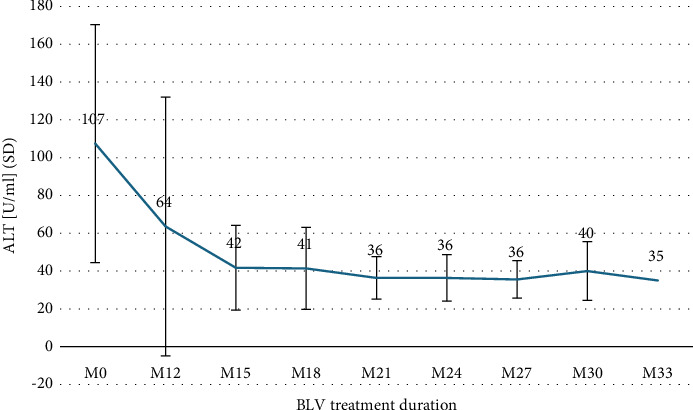
Biochemical response. SD: standard deviation; M: treatment months; BLV: bulevirtide.

The median duration of BLV therapy at the last follow-up (FU) was 26 months (IQR 17–27). Treatment duration was ≥12 months in 14 (100%) patients, ≥18 months in 10 (71%), and ≥24 months in 8 (57%). One patient had received BLV for 33 months. After the first 12 months of treatment, HDV-RNA decreased from log_10_ 5.58 IU/ml to log_10_ 3.01 IU/ml (SD 1.32) and ranged between log_10_ 2.19 IU/ml and log_10_ 3.19 IU/ml, thereafter (Figure 1). Changes in ALT levels during treatment are shown in Figure 2, and changes in VR, BR, and CR are shown in Table 2. After 12 months of treatment, there was only one (7%) patient with NR who then experienced VR after 15 months, and one (7%) patient with PR who had not yet reached the next FU. Two (25%) patients showed an increase in HDV-RNA >1 log_10_ IU/ml compared to previous viral load after 24 months of treatment but returned to previous HDV-RNA levels within the next FU. No effects on HBV-DNA and HBsAg were observed during the entire treatment period.

### 3.3. Adverse Events of Bulevirtid Treatment and Effect on Liver Function

**Figure 3:**
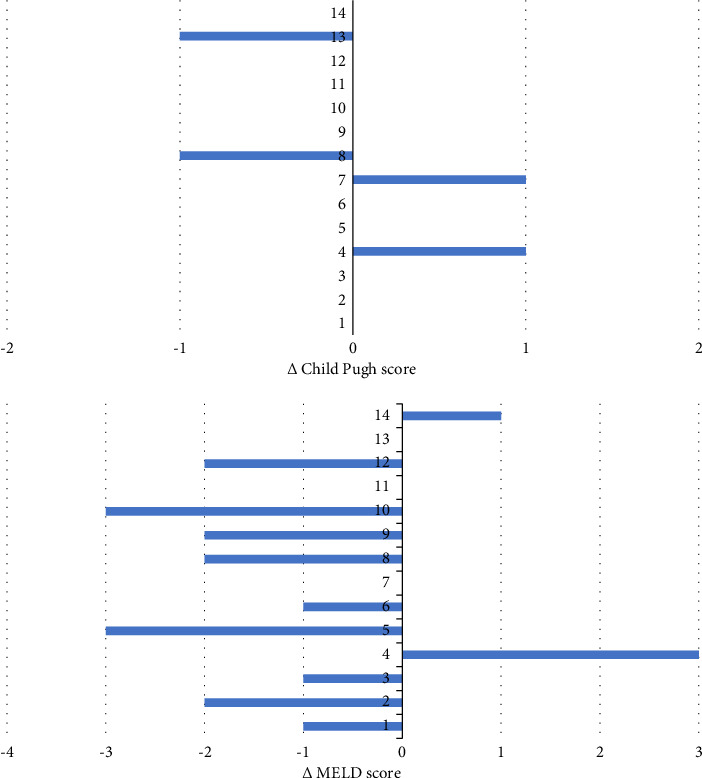
Changes in the Child Pugh score and MELD score between baseline and last follow-up. Each bar represents an individual patient. Negative values represent improvement, and positive values represent deterioration.

**Figure 4:**
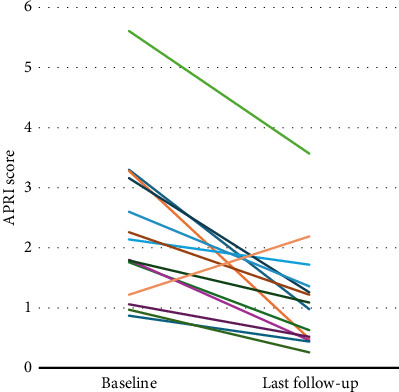
Changes in APRI score between baseline and last follow-up. Each line represents an individual patient.

Two patients reported mild and self-limiting adverse events (pruritus and abdominal discomfort), and treatment discontinuation was not necessary. Between baseline and last FU, the CP and MELD scores were maintained in 10 (71%) and 3 (21%) patients, improved in 2 (14%) and 9 (64%) patients, respectively, and worsened in 2 (14%) and 2 (14%) patients, respectively (Figure 3). The APRI at baseline was ≥1.09 in 11 (79%) patients, and the mean APRI significantly declined from 2.33 (SD 1.27) to 1.15 (SD 0.89) (*p* < 0.001) during treatment (Figure 4). Hepatic decompensation with ascites requiring large-volume paracentesis occurred in 1 (8%) patient with clinically significant portal hypertension after 24 months of treatment. At this time, the patient had a CR with HDV-RNA <LLQ, and there was no evidence of HCC or concomitant acute or chronic liver disease; therefore, BLV therapy was maintained. According to the BEA score, two (14%), eight (57%), and four (29%) patients were categorized as BEA-A, BEA-B, and BEA-C, respectively. The only decompensation event occurred in a patient classified as BEA-C during a cumulative FU of 319 months.

## 4. Discussion

In this retrospective monocentric study, BLV administration >12 months was associated with prolonged VR and BR as well as with preservation of liver function in patients with CHD and cACLD. Additionally, BLV was well tolerated and only a very limited number of side effects were observed.

CHD is a rare disease with a considerably lower prevalence than chronic hepatitis B and C [1, 18]. However, it is associated with a more aggressive course of liver disease and subsequently higher progression rates towards ACLD, portal hypertension, decompensation, and HCC [1, 3]. A preliminarily published German guideline on the treatment of CHD recommends evaluation of antiviral therapy in all patients with CHD with prioritization of patients with high inflammatory activity, advanced fibrosis, and compensated cirrhosis [19]. Until the conditional approval of BLV in July 2020, medical treatment options for CHD were mainly limited to PEG-IFN*α*-2a. Although PEG-IFN*α*-2a led to undetectable HDV-RNA in 24% and 33% after 48 and 96 weeks, respectively, half the patients experienced virological relapse after a median of 4.5 years [7, 8, 19], [20]. IFN treatment is also associated with frequent and occasionally severe adverse effects and has several contraindications. However, it was also associated with a lower risk of relevant clinical outcomes, such as hepatic decompensation and liver transplantation, compared to NA therapy alone [6]. Since persisting HDV viremia is a risk factor for cirrhosis and liver-related events, patients with CHD could benefit from viral suppression [21–23]. Hence, a decline in HDV-RNA of ≥2 log_10_ was suggested as an indicator of an effective treatment [24].

BLV was the first drug approved for CHD treatment. It results in a notable decrease in HDV-RNA levels by engaging with NTCP, thereby, hindering the infection of previously uninfected hepatocytes. Because BLV does not interfere with the HDV life cycle within infected hepatocytes, relapse of HDV-RNA is anticipated as soon as BLV treatment is stopped [11]. Therefore, BLV should be continued as long as a clinical benefit is expected [14]. Furthermore, VR seems to increase with treatment duration which may be explained by a decreased burden of infected hepatocytes [12]. Randomized controlled trials reported VR of 54% after 24 weeks and 71% after 48 weeks with BLV at 2 mg per day [11, 12]. In several real-world cohort studies, VR ranged between 58% and 78% after 24–48 weeks of treatment [25–31]. Due to its fairly recent approval, experience with long-term administration of BLV is limited. To address this issue, we included patients with treatment duration >12 months only in our investigation. Thus, higher rates of VR and HDV-RNA negativity in our study may be best explained by the mode of action of BLV, but also because of a positive selection bias. Rates for BR and CR were higher in some, but not all previously published studies [25–31]. We observed corresponding rates for VB, which occurred in 9–14% [27, 28]. Both cases with VB showed significant reduction in HDV-RNA at the next FU visit. In these patients, bile acids were not available to evaluate treatment adherence. In line with previous studies, we did not observe changes in quantitative HBsAg levels during BLV treatment [27, 32].

Despite frequent reporting of adverse events (AEs) in randomized controlled trials (64–82%), AE grade 3 or 4 and serious AE were infrequent (10–11% and 0–4%, respectively) in BLV 2 mg treatment groups [11, 12]. Importantly, treatment discontinuation due to AE was not observed in our study. The most common AEs in the MYR301-trial, a phase 3 study, were headache, pruritus, fatigue, nausea, arthralgia, and injection-site events, e.g., erythema, pruritus, and swelling as well as changes in laboratory values [12]. In our analysis, BLV was well tolerated even during long-term administration in our study with no injection-site event, no serious AE, and no treatment discontinuation. The favorable safety profile of BLV could be attributed to its metabolism. As a peptide, BLV is likely degraded by proteases into amino acids without involvement of cytochrome enzymes [33]. Additionally, no active metabolites are expected.

Portal hypertension is a key determinant for the risk of hepatic decompensation in patients with ACLD and can be estimated by non-invasive testing, e.g., liver stiffness measurement (LSM) [34]. The majority of patients in our study had assumed or suggested cACLD with a corresponding high prevalence of portal hypertension. Accordingly, most patients had an APRI ≥1.09 at baseline, which had a positive predictive value of 85% for a hepatic venous pressure gradient >12 mmHg [35]. Whereas liver stiffness measurements were not regularly available during FU, we observed a decrease in APRI in all but one patient and a significant reduction in mean APRI. However, changes in APRI were mainly driven by changes in AST levels and not by an increase or decrease in platelet count. Therefore, it is questionable if improvements in APRI represent true improvements in portal hypertension or if this is due to a reduction in hepatic inflammation. The single patient with an increased APRI, who had only PR after 12 months of treatment, also experienced an increase in liver stiffness from 9 kPa to 14 kPa, which was confirmed on separate occasion. Degasperi et al., however, described similar findings with improvement in AST and unchanged platelet count after 48 weeks of BLV treatment in patients with ACLD and, additionally, a significant decrease in liver stiffness [30].

An important finding of our study is that liver function, represented by Child Pugh and MELD scores, was stable or improved in the majority of patients during long-term treatment with BLV. Only a single case of hepatic decompensation in terms of ascites requiring large-volume paracentesis was observed in one patient during the entire FU. MELD score improved more frequently than Child Pugh score which may be best explained by slight improvements in laboratory values (i.e., bilirubin, creatinine, INR, and albumin), which was sufficient to improve MELD but not Child Pugh score, which categorizes liver function more broadly.

Albeit comprising a shorter FU, increased albumin, stable bilirubin, and no decompensating event or HCC were reported in the study by Degasperi et al., emphasizing the reliability of our results [30]. The BEA score was developed to predict the probability of liver-related events in patients with CHD, and to stratify patients into three different risk categories [17]. In the development cohort, patients with BEA scores B and C had a hazard ratio of 9 and 25, respectively, for liver-related events compared to patients with BEA score A [17]. According to the BEA score, the majority of our patients were categorized as having a moderate and high risk, for whom a higher incidence of hepatic events was anticipated than was actually observed. Although we did not perform survival analysis because of the low patient numbers and relatively short FU for this purpose, our results may suggest that treatment with BLV stabilizes liver function and reduces the risk of liver-related events.

This study has several limitations, which are mainly attributed to the small cohort size and monocentric and retrospective design. On the other hand, our study comprised a relatively long follow-up period (up to 33 months) and a relatively large cohort of patients with CHD and ACLD in comparison to other published real-world experiences with BLV.

In conclusion, treatment with bulevirtide beyond one year appears to be effective and safe in patients with chronic hepatitis D and advanced chronic liver disease. While only one case of hepatic decompensation occurred during a median FU of 26 months, liver function was stable or even improved during treatment in the majority of patients.

## Figures and Tables

**Table 1:** Baseline characteristics.

Gender, *n* (%)	Male	9 (64)
	Female	5 (36)

Age, median (IQR)	Years	48 (37–55)

HBe antigen, *n* (%)	Negative	12 (86)
	Positive	2 (14)

HBV-DNA < LLQ	No	3 (21)
	Yes	11 (79)

HBs antigen, mean (SD)	IU/ml	13.234 (12.567)

HDV-RNA, mean log_10_ (SD)	IU/ml	5.58 (1.08)

Concomitant NA, *n* (%)	No	0 (0)
	Yes	14 (100)

Prior IFN, *n* (%)	No	8 (57)
	Yes	6 (43)

ACLD	No	2 (14)
	Yes	12 (86)

Prior decompensation, *n* (%)	No	12 (86)
	Yes	2 (14)

Varices, *n* (%)	No	9 (64)
	Yes	5 (36)

Ascites, *n* (%)	No	13 (93)
	Yes	1 (7)

HE, *n* (%)	No	14 (100)
	Yes	0 (0)

Splenomegaly, *n* (%)	No	3 (21)
	Yes	11 (79)

Thrombocytopenia, *n* (%)	No	2 (14)
	Yes	12 (86)

Child Pugh score, *n* (%)	A	13 (93)
	B	1 (7)
	C	0 (0)

MELD, median (IQR)		9 (7–10)

Liver stiffness, mean (SD)	kPa	17.1 (7.4)

Platelets, mean (SD)	k/*µ*l	98 (39)

ALT, mean (SD)	U/l	107 (63)

AST, mean (SD)	U/l	85 (36)

GGT, mean (SD)	U/l	86 (63)

Bilirubin, mean (SD)	mg/dl	1.1 (0.5)

Creatinine, mean (SD)	mg/dl	0.79 (0.14)

INR, mean (SD)		1.19 (0.11)

Albumin, mean (SD)	g/dl	40 (6)

IQR: interquartile range; SD: standard deviation; LLQ: lower limit of quantification; NA: nucleos(t)ide-analogue therapy; IFN: interferon; ACLD: advanced chronic liver disease; HE: hepatic encephalopathy; MELD: model of end-stage liver disease; ALT: alanine aminotransferase; AST: aspartate aminotransferase; GGT: gamma-glutamyltransferase; INR: international normalized ratio.

**Table 2:** Virological (VR), biochemical (BR), and combined response (CR) depending on time after start of bulevirtide; T: treatment month; LLQ: lower limit of quantification.

	T12	T15	T18	T21	T24	T27	T30	T33
VR (%)	86	100	90	100	75	100	100	100
HDV-RNA < LLQ (%)	43	50	50	57	63	57	0	0
BR (%)	43	60	60	86	75	86	50	100
CR (%)	43	60	60	86	75	86	50	100
Patients (*n*)	14	10	10	7⁣^*∗*^	8	7	2	1

⁣^*∗*^In one patient, who has been treated for 24 months, data for T21 were not available.

## Data Availability

The retrospectively collected patient data used to support the findings of this study are available from the corresponding author upon request.

## Abbreviations

HDV: Hepatitis delta virus
HBsAg: Hepatitis B surface antigen
HCC: Hepatocellular carcinoma
CHD: Chronic hepatitis D
HBV: Hepatitis B virus
NTCP: Sodium taurocholate cotransporting polypeptide
PEG-IFN*α*: Pegylated interferon alfa
NA: Nucleos(t)ide-analogue
BLV: Bulevirtide
EMA: European Medicines Agency
ACLD: Advanced chronic liver disease
ALT: Alanine aminotransferase
MELD: Model of end-stage liver disease
AST: Aspartate aminotransferase
APRI: Aspartate aminotransferase-to-platelet-ratio-index
D4FS: Delta-4 fibrosis score
BEA: Baseline-event anticipation
SD: Standard deviation
IQR: Interquartile range
LLQ: Lower limit of quantification
FU: Follow-up.

## Additional Points

*Lay Summary*. Bulevirtide, which is approved for the treatment of chronic hepatitis D, led to an effective and sustained reduction in hepatitis D viremia in patients with chronic hepatitis D and advanced chronic liver disease. In this retrospective analysis, liver function was stable or improved in most patients without severe adverse effects during long-term treatment with bulevirtide.
